# Bevacizumab plus capecitabine as later-line treatment for patients with metastatic colorectal cancer refractory to irinotecan, oxaliplatin, and fluoropyrimidines

**DOI:** 10.1038/s41598-021-86482-x

**Published:** 2021-03-29

**Authors:** Yeong Hak Bang, Jeong Eun Kim, Ji Sung Lee, Sun Young Kim, Kyu-Pyo Kim, Tae Won Kim, Yong Sang Hong

**Affiliations:** 1 Department of Oncology, Asan Medical Center, University of Ulsan College of Medicine, 88 Olympic-ro 43-gil, Songpa-gu, Seoul, 05505 Korea; 2 Clinical Research Center, Asan Institute for Life Sciences, University of Ulsan College of Medicine, Seoul, Korea; 3 Department of Digital Health, Samsung Advanced Institute for Health Science and Technology, Sungkyunkwan University, Seoul, Korea

**Keywords:** Gastrointestinal cancer, Cancer therapy

## Abstract

There is an unmet medical need for later-line treatment options for patients with metastatic colorectal cancer (mCRC). Considering that, beyond progression, co-treatment with bevacizumab and cytotoxic chemotherapy showed less toxicity and a significant disease control rate, we aimed to evaluate the efficacy of capecitabine and bevacizumab. This single-center retrospective study included 157 patients between May 2011 and February 2018, who received bevacizumab plus capecitabine as later-line chemotherapy after progressing with irinotecan, oxaliplatin, and fluoropyrimidines. The study treatment consisted of bevacizumab 7.5 mg/kg on day 1 and capecitabine 1,250 mg/m^2^ orally (PO) twice daily on day 1 to 14, repeated every 3 weeks. The primary endpoint was progression-free survival (PFS). The median PFS was 4.6 months (95% confidence interval [CI] 3.9–5.3). The median overall survival (OS) was 9.7 months (95% CI 8.3–11.1). The overall response rate was 14% (22/157). Patients who had not received prior targeted agents showed better survival outcomes in the multivariable analysis of OS (hazard ratio [HR] = 0.59, 95% CI 0.43–0.82, *P* = 0.002) and PFS (HR = 0.61, 95% CI 0.43–0.85, *P* = 0.004). Bevacizumab plus capecitabine could be a considerably efficacious option for patients with mCRC refractory to prior standard treatments.

## Introduction

Metastatic colorectal cancer (mCRC) is one of the most common causes of cancer-related death^1^. In first- or second-line treatment, the addition of targeted drugs consisting of anti-vascular endothelial growth factor (VEGF) agents (bevacizumab, aflibercept, and ramucirumab) and anti-epidermal growth factor receptor (EGFR) agents (cetuximab and panitumumab) to oxaliplatin or irinotecan doublets improved survival outcomes with median overall survival more than 2 years^2–8^.

Recent studies on later-line treatments showed promising efficacy among mCRC patients harbouring the following specific genetic alterations: amplified human epidermal growth factor receptor 2 (HER2)^9^, neurotrophic receptor tyrosine kinase (NTRK) fusion^10^, mutated B-Raf proto-oncogene, serine/threonine kinase (BRAF)^11,12^, and mismatch repair deficiency^13–15^. These newer agents are currently under trial for first-line or second-line rather than later-line treatment administration. However, the incidence of these specific alterations is very low (< 5%) and is not always applicable. Moreover, the different reimbursement policies used for diagnosis and treatment by some countries limit the administration of newer agents to certain patients.

For most patients without these specific target alterations described above, third- or later-line treatment consists of regorafenib or TAS-102—an orally available, small molecular multi-kinase inhibitor and a compound of trifluridine with tipiracil, respectively—which showed survival benefits in a randomised controlled trial^16–18^. However, the numerical increase in survival duration was < 3 months compared with the placebo. In addition, the aforementioned later-line treatment options only benefit a limited number of patients because of the high cost. Consequently, there is an unmet medical need for easily accessible later-line treatment options with minimal costs.

Capecitabine monotherapy is not recommended for later-line treatment, especially in those who have progressed after treatment with fluoropyridine plus oxaliplatin or irinotecan doublets. However, it has been commonly used in the later-line treatment of patients with refractory mCRC, though the supporting evidence is weak^19^. Bevacizumab for later-line treatment is not recommended, even for those who were not exposed to first- or second-line treatment^20^.

Continuation of bevacizumab beyond disease progression with cytotoxic agents and bevacizumab co-therapy showed clinical benefit in patients with mCRC^21,22^. These studies suggest that bevacizumab therapy after disease progression may have clinical benefit, even in refractory patients treated with bevacizumab. A recent study demonstrated that bevacizumab plus TAS-102 as later-line treatment improved survival rates^23^. Therefore, we aimed to evaluate the efficacy of the combination of bevacizumab and capecitabine.

## Patients and methods

### Patients and data collection

We retrospectively accrued 157 mCRC patients who received bevacizumab plus capecitabine as their third- or later-line treatment in Asan Medical Center, Korea, between May 2011 and February 2018. Patients were intolerant or progressed during or within 6 months of the standard irinotecan, oxaliplatin, and fluoropyrimidine therapies. Patients who were previously administered capecitabine were included. The study treatment, which consisted of bevacizumab 7.5 mg/kg on day 1 and capecitabine 1,250 mg/m^2^ orally (PO) twice daily on day 1 to 14, was repeated every 3 weeks.

Clinical data were obtained from medical records using Asan Biomedical Research Environment (ABLE), an anonymous clinical database system in our institute. The primary endpoint was progression-free survival (PFS) and the secondary endpoints were overall survival (OS), confirmed best overall response, safety outcomes, and a risk factor affecting survival outcomes. For the safety evaluation, we analysed the number and proportion of patients who experienced any events according to the common terminology criteria for adverse events (CTCAE) version 4.03.

To analyse the best overall response, we used the response evaluation criteria in solid tumours (RECIST) version 1.1. We investigate the clinical characteristics of patients who showed an extended lasting response with a combination of bevacizumab and capecitabine. The study was conducted in accordance with the Declaration of Helsinki, and the protocol was approved by the Institutional Review Boards (IRBs) of Asan Medical Center, Korea (approval number: 2020–1875). The requirement of obtaining informed consent from patients was waived by the IRBs because of the retrospective nature of this study.

### Statistical Analysis

Patients’ demographics, clinicopathological characteristics, and treatment patterns were summarised as numbers (percentages) for categorical variables and means for continuous variables. Survival curves were constructed using paired Kaplan–Meier estimates and analysed using the stratified log-rank test. Univariable and multivariable analysis of PFS and OS were performed using the Cox proportional hazards model. The variables were age, sex, primary site, treatment lines, RAS mutation, previous history of targeted agents, and time from diagnosis of metastatic disease to treatment. In the multivariable analysis, variables with a potential relationship (*P* < 0.2) in the univariable analyses were included. All reported P-values were two-sided, and a *P* < 0.05 was considered statistically significant.

## Results

### Patient population and treatment summary

The baseline characteristics of the 157 patients are shown in Table 1. The median age was 57 (range, 28–82) years and more than half of the patients had two or more metastatic sites, whereas 33 (21.0%) patients experienced peritoneal seeding. More than half (52.9%) and eight (5.1%) patients had tumours with RAS and BRAF mutations, respectively. Among the patients with RAS and BRAF wild-type, 52 had a history of treatment with targeted agents before capecitabine and bevacizumab, 19 (27.1%) received both bevacizumab and cetuximab, one (1.4%) had bevacizumab without cetuximab, and 32 (45.7%) received only cetuximab treatment.

**Table 1:** Baseline characteristics of all patients (N = 157).

	N	%
**Age, years**		
< 65	116	73.9
≥ 65	41	26.1
**Sex**		
Male	85	54.1
Female	72	45.9
**Primary site**		
Right colon	42	26.8
Left colon	115	73.2
**Histology**		
W/D adenocarcinoma*	10	6.4
M/D adenocarcinoma*	118	75.2
P/D adenocarcinoma*	29	18.5
**Site of metastasis**		
Liver	91	58.0
Lung	62	39.5
Lymph node	50	31.8
Peritoneum	33	21.0
Others*	20	12.7
**Sum of metastasis**		
1 organ metastasis, excluding peritoneum	65	41.4
2 or more organ metastasis, excluding peritoneum	59	37.6
Any number of organs plus peritoneum	33	21.0
**RAS mutation**		
Wild	74	47.1
Mutant	83	52.9
**BRAF mutation**		
Wild	142	90.4
Mutant	8	5.1
Unknown	7	4.5
**Previous systemic treatment lines**		
2	85	54.1
≥ 3	72	45.9
**Time from first diagnosis of metastatic disease to treatment**		
< 24 months	74	47.1
≥ 24 months	83	52.9
**Previous targeted agents**		
RAS/RAF wild-type (n = 70)		
Prior bevacizumab and cetuximab	19	12.1
Prior bevacizumab	1	0.1
Prior cetuximab	32	20.4
RAS or RAF mutated (n = 87)		
Prior bevacizumab	36	22.9
Previous chemotherapy		
**Fluoropyrimidine**		
Refractory	157	100
Intolerant	0	0
**Oxaliplatin**		
Refractory	149	96.1
Intolerant	4	2.5
**Irinotecan**		
Refractory	156	99.4
Intolerant	1	0.6

*Ovary 4%, bone 8%, etc., 4%.

*W/D* well differentiated, *M/D* moderately differentiated, *P/D* poorly differentiated, *MSI* microsatellite instability, *MSS* microsatellite stable. *VEGF* vascular endothelial growth factor, *EGFR* epidermal growth factor receptor.

FOLFOX; FU, leucovorin, and oxaliplatin, FOLFIRI; fluorouracil (FU), leucovorin, and irinotecan.

Among the 87 patients who were RAS or BRAF mutant, 36 (41.4%) were administered bevacizumab before treatment. Fifty-six (35.7%) of the 157 patients did not receive bevacizumab as their first- or second-line treatment and 8 (5.1%) were already exposed to capecitabine before the study treatment. The treatment exposures of the patients are summarised in Table 2. Patients received a median of six cycles of bevacizumab and capecitabine.

**Table 2:** Treatment exposure (safety population).

Total number of cycles administered (median), [range], [IQR]	1,079 (6) [Range, 1–31], [IQR 3–9]
Cycles with delayed schedule	33(3.1%)
**Cycles with dose modification were made**	
Capecitabine	776 (71.9%)
Bevacizumab	10(0.9%)
**Relative dose intensity, median (%), [range], [IQR]**	
Capecitabine (twice per day, mg/m^2^)	80.2% (1,003 mg/m^2^ [Range, 778.4–1282.1], [IQR 926.5–1227]
Bevacizumab (mg/kg)	100% (7.5 mg/kg) [Range, 5.0–7.5], [IQR 7.5–7.5]

The median dose intensities of capecitabine and bevacizumab were 80.2% (1,003 mg/m^2^ PO twice daily, interquartile range [IQR], 74.1%–98.2%) and 100% (7.5 mg/kg, IQR, 100%–100%), respectively. A total of l079 cycles of treatment were administered, including 776 (71.9%) and 10 (0.9%) cycles with dose modifications made for capecitabine and bevacizumab, respectively. The median follow-up duration was 7.9 (IQR, 4.3–15.1) months.

### Response to treatment and survival

**Figure 1:**
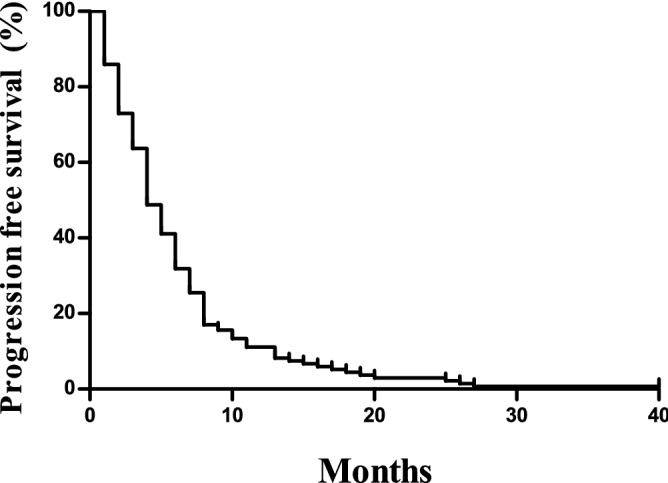
Progression free survival of the study cohort.

**Figure 2:**
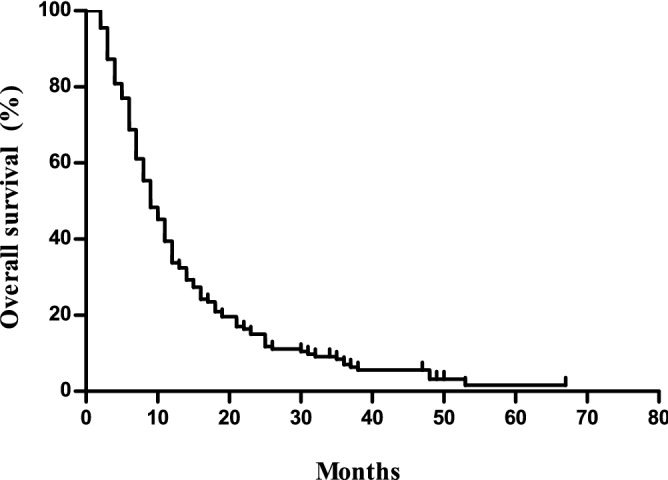
Overall Survival of the study cohort.

The median PFS was 4.6 (95% confidence interval [CI] 3.9–5.3) months, and the median OS was 9.7 (95% CI 8.3–11.1) months (Figs. 1 and 2). The overall response rate (ORR) was 14.0% with 22 (14.0%), 83 (52.9%), and 43 (27.4%) patients showing partial responses, stable disease, and progressive disease, respectively, based on the intent-to-treat analysis. The response was not evaluated in nine patients (5.7%). Table 3 summarises the univariable and multivariable analysis of the PFS and OS.

**Table 3:** Multivariable analysis using the Cox regression model for survival outcomes.

	PFS				OS			
	Crude		Adjusted*		Crude		Adjusted**	
	HR (95% CI)	*P*-value	HR (95% CI)	*P*-value	HR (95% CI)	*P*-value	HR (95% CI)	**P-value**
**Age, years**								
< 65 (n = 116)	Reference	0.926			Reference	0.315		
≥ 65 (n = 41)	0.99 (0.82–1.19)				1.21 (0.84–1.74)			
**Sex**								
Male (n = 85)	Reference	0.148	Reference	0.025	Reference	0.519		
Female (n = 72)	0.78 (0.56 –1.10)		0.67 (0.48 –0.95)		0.90 (0.65–1.24)			
**Primary site**								
Rt. colon (n = 42)	Reference	0.079	Reference	0.214	Reference	0.126	Reference	0.147
Lt. colon (n = 115)	1.40 (0.96–2.04)		1.27 (0.87–1.87)		1.33 (0.92–1.92)		1.33 (0.90–1.96)	
**Previous treatment lines**								
2 (n = 84)	Reference	0.133	Reference	0.328	Reference	0.212		
≥ 3 (n = 73)	1.29 (0.93–1.79)		1.20 (0.83–1.75)		1.23 (0.89–1.70)			
**RAS mutation**								
Wild (n = 74)	Reference	0.015	Reference	0.125	Reference	0.011	Reference	0.594
Mutant (n = 83)	0.66 (0.47–0.91)		0.75 (0.51–1.09)		0.66 (0.48–0.91)		0.77 (0.55–1.09)	
**Previous targeted biological treatment**								
Yes (n = 87)	Reference	0.011	Reference	0.004	Reference	0.002	Reference	0.002
No (n = 70)	0.65 (1.10–2.14)		0.61 (0.43–0.85)		0.59 (0.42–0.82)		0.59 (0.43–0.82)	
**Time from first diagnosis of metastatic disease to treatment**								
< 24 months (n = 74)	Reference	0.043	Reference	0.018	Reference	0.154	Reference	0.103
≥ 24 months (n = 83)	0.71 (0.51–0.99)		0.67 (0.48–0.93)		0.79 (0.57–1.09)		0.76 (0.55–1.06)	

*Adjusted for sex, primary site, prior chemotherapy lines, RAS mutation, prior history of targeted agents, and time from diagnosis of metastatic disease to treatment.

**Adjusted for primary site, RAS mutation, prior history of targeted agents, and time from diagnosis of metastatic disease to treatment.

After the multivariable analysis, female sex (hazard ratio [HR], 0.67; 95% CI 0.48–0.95; *P* = 0.025), no prior history of targeted biologic treatment (HR, 0.61; 95% CI 0.43–0.85; *P* = 0.004), the interval between first diagnosis of metastatic disease to treatment (≥ 24 months; HR, 0.71; 95% CI 0.48–0.93; *P* = 0.018) were significantly associated with a longer PFS. Only no prior history of treatment with targeted agents was associated with a longer OS (HR, 0.59; 95% CI 0.43–0.82, *P* = 0.002).

**Figure 3:**
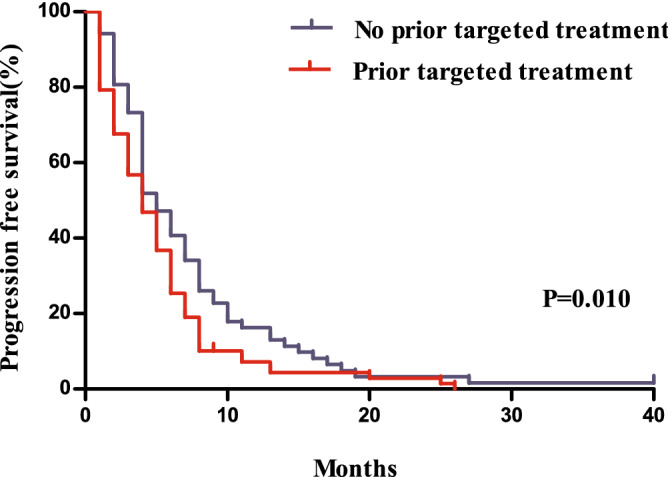
Comparison of progression free survivals between patients with and without a history of the administration of targeted agents.

**Figure 4:**
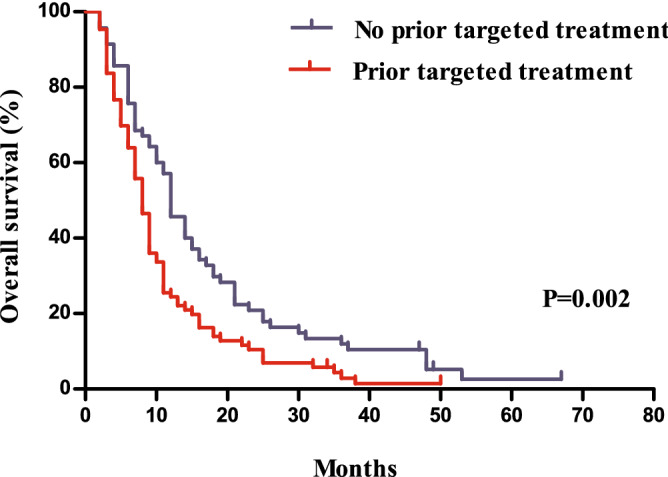
Comparison of overall survivals between patients with and without a history of the administration of targeted agents.

**Figure 5:**
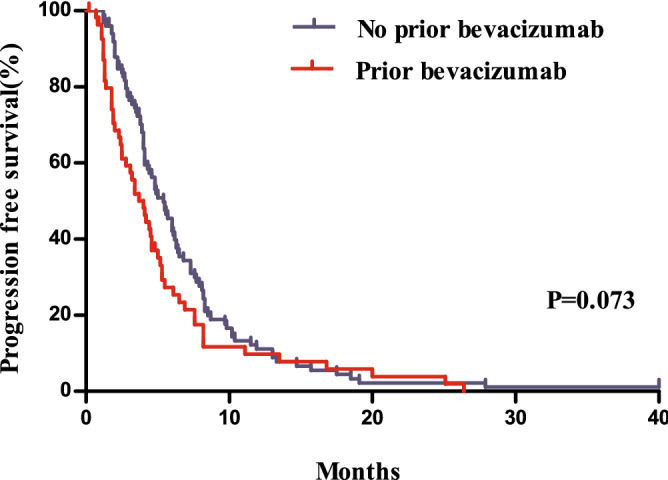
Comparison of progression free survivals between patients with and without a history of bevacizumab treatment.

**Figure 6:**
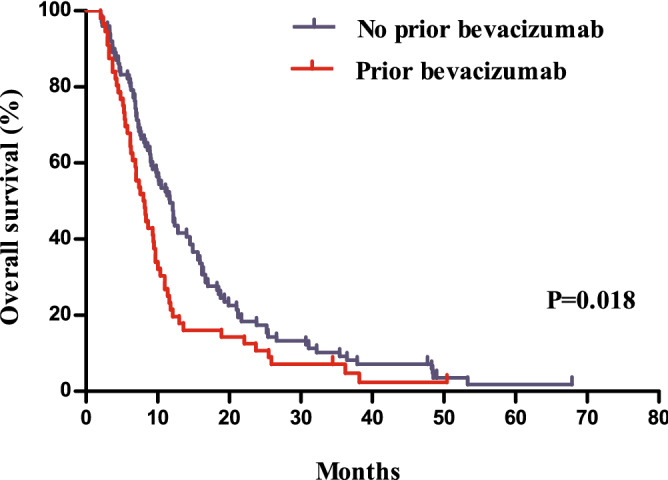
Comparison of overall survivals between patients with and without a history of bevacizumab treatment.

Compared to patients with a prior history of receiving targeted agents, those without showed a significantly longer PFS (*P* = 0.010, median 5.0 months vs. 4.1 months) and OS (*P* = 0.002, median 12.2 months vs. 8.2 months, Figs. 3 and 4). Patients without a history of treatment with bevacizumab showed PFS that was not significantly different from that of patients with a history of bevacizumab treatment (*P* = 0.073, 5.4 months vs. 4.0 months), whereas they showed a better ORR (one-sided *P* = 0.015, 19 [18.8%] vs. 3 [5.4%]) and significantly longer OS (*P* = 0.018, 11.7 months vs. 8.0 months, Figs. 5 and 6).

### Safety profile of co-treatment with bevacizumab and capecitabine

The incidence of adverse events following co-treatment with bevacizumab and capecitabine is summarised in Table 4. The most common toxicity symptom was hand-foot syndrome (58.6%) and approximately 5% of patients showed a grade 3 level. Ten (6.4%) and 11 (7.0%) patients showed proteinuria and bleeding events, respectively. Moreover, there was no thromboembolic events among the patients. Notably, 14 patients had to stop chemotherapy because of toxicity, 2 discontinued because of bleeding events, 3 had severe proteinuria, 1 experiences perforation of the bowel, and 8 refused chemotherapy because of poor tolerance.

**Table 4:** Adverse event after bevacizumab/capecitabine treatment.

Adverse events	Any grade	Grade 3 or 4
Any event	157 (100%)	28 (17.8%)
Hand foot syndrome	92 (58.6%)	8 (5.1%)
Fatigue	24 (15.3%)	2 (1.3%)
Nausea	14 (8.9%)	4 (2.6%)
Vomiting	8 (5.1%)	3 (1.9%)
Mucositis	35 (22.3%)	2 (1.3%)
Diarrhoea	16 (10.2%)	1 (0.6%)
Neutropenia	39 (24.8%)	5 (3.2%)
Thrombocytopenia	54 (34.4%)	2 (1.3%)
Anaemia	96 (61.1%)	2 (1.3%)
LFT elevation	76 (48.4%)	2 (1.3%)
Bilirubin elevation	64 (40.8%)	5 (3.2%)
Proteinuria	10 (6.4%)	3 (1.9%)
Bleeding/haemorrhage	11 (7.0%)	2 (1.3%)
Gastrointestinal perforation	1 (0.6%)	1 (0.6%)
Thromboembolic event	0 (0.0%)	0 (0.0%)

## Discussion

In this study, co-treatment with bevacizumab and capecitabine in patients with mCRC refractory to at least two chemotherapy regimens showed efficacy with a 65% disease control rate, including a 14% PR, whereas the PFS and OS after treatment were 4.6 months and 9.7 months, respectively. This result indicted that the efficacy and treatment outcomes were comparable to those of previous studies^16–18,24^.

A previous study evaluating the efficacy of single-agent capecitabine in 5-FU resistant mCRC did not show a significant clinical benefit^25^. However, another study that assessed the effectiveness of co-treatment with bevacizumab and capecitabine after second-line treatment in 34 mCRC patients showed efficacy with disease control in most patients, with partial responses and stable disease in 3 (9%) and 21 (62%) patients, respectively. The median PFS and OS were 5.4 months and 12.2 months, respectively. Only hypertension was a common grade > 2 toxicity (24%), and none of the patients developed grade 3 or 4 bleeding, thrombosis, or intestinal perforation^26^. Although this study was small-sized, the results showed better survival benefits than those shown by other third-line data, with less toxicity. In our research, more patients with mCRC were included and they showed similar response rates and toxicities. Despite the shorter median OS and PFS, our results still showed comparable survival benefits with those of other third-line studies.

Co-treatment with new fluoropyrimidine and bevacizumab was also effective in refractory mCRC patients in recent studies. In a real-world study of TAS-102 with or without bevacizumab treatment in 57 refractory mCRC patients, co-treatment showed longer survival, with an OS of 14.4 months in the TAS-102 plus bevacizumab group and 6.1 months in the TAS-102 monotherapy group (HR, 0.33; 95% CI 0.15–0.73; *P* = 0.006^27^. Furthermore, a recent phase 2 randomised controlled study showed that co-treatment with TAS-102 and bevacizumab was associated with clinically relevant significantly longer survival than TAS-102 monotherapy without increased toxicity; The OS was 9.4 months and 6.7 months in the TAS-102 plus bevacizumab and TAS-102 monotherapy groups (HR, 0.55; 95% CI 0.32–0.94; *P* = 0.028^23^.

Regorafenib or TAS-102 showed high frequency of toxicity in previous studies. For regorafenib, 54% of patients showed ≥ grade 3 adverse events (16% hand-foot reaction, 11% hypertension) in the CONCUR study^17^. TAS 102 also induced grade 3 adverse events in 45.8% of patients in the TERRA study^28^. Compared to these agents, co-treatment with bevacizumab and capecitabine showed fewer incidences of ≥ grade 3 adverse events in our study. Specifically, 28 (17.8%) patients showed ≥ grade 3 adverse events and 14 (8.9%) had to stop chemotherapy because of the adverse events. Although our study was retrospective, co-treatment with bevacizumab and capecitabine could be a tolerable treatment option.

Recently, TASCO study compared the TAS-102 plus bevacizumab with capecitabine plus bevacizumab as the first-line treatment for patients with mCRC ineligible for intensive therapy. Although there was no statistically significant difference, the TAS-102 plus bevacizumab showed better clinical outcomes compared to capecitabine with median PFS 9.2 and 7.8 months (HR, 0.71;95% CI 0.48–1.06) and median OS was 22.3 and 17.7 months (HR, 0.78; 95% CI 0.55–1.10) respectively. Though this study was targeting different treatment settings from our study, the TAS-102 plus bevacizumab is likely to be the superior regimen to capecitabine and bevacizumab. On the other hand, TAS-102 plus bevacizumab regimen showed more grade 3 hematologic events such as decreased neutrophil count than capecitabine plus bevacizumab (18% vs. 1%). Considering hematologic toxicity and accessibility of TAS-102, the combination of bevacizumab and capecitabine also could be a good later line option^29,30^.

This study has some limitations, such as the single-centre retrospective design, absence of a control group, and the sample size, which was too small to verify the effectiveness of treatment. Nevertheless, our research showed the therapeutic efficacy and tolerability of bevacizumab plus capecitabine beyond second-line treatment in mCRC patients who had two or more lines of chemotherapies. This was especially evident in patients who had not previously used targeted agents. We consider that the combination bevacizumab and capecitabine regimen could be a useful treatment option for patients who are refractory to later-line treatments in mCRC.
